# Data-Gathering Scheme Using AUVs in Large-Scale Underwater Sensor Networks: A Multihop Approach

**DOI:** 10.3390/s16101626

**Published:** 2016-09-30

**Authors:** Jawaad Ullah Khan, Ho-Shin Cho

**Affiliations:** School of Electronics Engineering, Kyungpook National University, Daegu 702-701, Korea; jawaad_khan@ee.knu.ac.kr

**Keywords:** Underwater Sensor Networks, Autonomous Underwater Vehicle, Voronoi region, tour-point, data-gathering

## Abstract

In this paper, we propose a data-gathering scheme for hierarchical underwater sensor networks, where multiple Autonomous Underwater Vehicles (AUVs) are deployed over large-scale coverage areas. The deployed AUVs constitute an intermittently connected multihop network through inter-AUV synchronization (in this paper, *synchronization* means an interconnection between nodes for communication) for forwarding data to the designated sink. In such a scenario, the performance of the multihop communication depends upon the synchronization among the vehicles. The mobility parameters of the vehicles vary continuously because of the constantly changing underwater currents. The variations in the AUV mobility parameters reduce the inter-AUV synchronization frequency contributing to delays in the multihop communication. The proposed scheme improves the AUV synchronization frequency by permitting neighboring AUVs to share their status information via a pre-selected node called an agent-node at the static layer of the network. We evaluate the proposed scheme in terms of the AUV synchronization frequency, vertical delay (node→AUV), horizontal delay (AUV→AUV), end-to-end delay, and the packet loss ratio. Simulation results show that the proposed scheme significantly reduces the aforementioned delays without the synchronization time-out process employed in conventional works.

## 1. Introduction and Motivation

With the consistent growth in human population, the demand for the exploration of new venues, where abundant resources required for human existence can be found, has increased proportionally over the last few decades. The vast expanses of the oceans constitute such unexplored venues and are considered as reservoirs of energy and food resources. For this purpose, researchers need to continuously collect oceanographic data for the exploration of the immense varieties of minerals and fuel over vast surveying areas and for monitoring the changes that affect the marine ecosystem for observing the evolution of the food resources. For such data collection, sensors are usually deployed over a large field and the collected data is periodically retrieved by visiting each sensor in the field. This conventional approach of offshore exploration and ecosystem monitoring becomes expensive and time-consuming when the surveying area increases. Therefore, most researchers are striving for the development of new exploration techniques and technologies to reduce the operational cost and time for such offshore exploration and monitoring. One of the most promising solutions is the usage of real-time communication between the sensors and the control center [1]. The real-time communications between the sensors and the control center constitute an underwater sensor network (UWSN) that meets the cost effectiveness and time efficiency requirements of the marine applications. In most of these networks, reliable two-way links between the nodes are formed using acoustic technology to overcome severe fading conditions and achieve a long-range communication. Most of the installed nodes are battery powered; hence, they have very limited energy resources. Therefore, the nodes are forced to transmit at short ranges to avoid a high power consumption and unnecessary interference with the ongoing communication over the network that may result in unnecessary retransmissions for the nodes. In a networking scenario where large numbers of nodes are deployed, some of the nodes deployed at a distance from the destination have to employ the services of other nodes for efficiently forwarding the data resulting in multihop transmissions over the network. This may increase the rate of utilization of the energy resources for the nodes located close the destination. This unequal rate of energy consumption results in an energy-hole problem for the network [2]. Therefore, various data forwarding schemes that enable the multihop transmissions over the sensor field for delivering the data to the destination and for overcoming uneven energy consumption by controlling certain transmission parameters and forwarding paths [3,4,5] have been presented. However, these protocols perform poorly in large-scale networks, where the usage of additional bandwidth and energy resources are required for the end-to-end route maintenance in long-term applications.

In [6,7,8,9,10], several schemes have been proposed, wherein a mobile node such as an AUV acts as a relay node to collect data from the normal sensors. In these schemes, the mobile node uses a specific trajectory called the “*tour-path*” to traverse the network. It stops at a specific location called the “*tour-point*” to retrieve the gathered data from the static normal nodes in a neighborhood. Even though the proposed schemes are able to mitigate the unequal energy consumption, they become ineffective for large-scale networks, where the mobile node trajectories are relatively large; accordingly, considerable data-gathering latency and expensive operational costs are incurred. Further, owing to the constraints of the on-board energy resources, the operational time of a mobile node is limited, consequently reducing the coverage area. The operational problems faced by a mobile node can be addressed if multiple mobile nodes are employed, where each mobile traverses the network on a distinct trajectory [11,12,13,14,15,16]. Then, these mobile nodes may coordinate with each other to remain in constant interconnection, enabling multihop communications over the coverage area. However, the constantly changing underwater conditions and AUVs’ mobility make it impossible to achieve continuous interconnection among the neighboring AUVs.

Under such an imperfect communication scenario, it is assumed that a certain configuration of mobile nodes leads to better coordination. This results in an “on/off” communication model, where multiple AUVs are assumed to be not in constant interconnection owing to the topological changes caused by the varying medium and the AUVs’ mobility [15]. In such scenarios, the AUVs behave like an intermittently connected network (ICN). In [16], authors presented a multiple AUV scheme using the concept of AUVs’ synchronization at agreed locations called rendezvous points (RPs) to achieve multihop communication. Here, two nodes are said to be synchronized when they are connected to each other for communication. The coverage area is divided into a number of regions called lanes wherein an AUV travels on a tour-path and stops at predefined RPs to synchronize with the neighboring AUVs. At the RP, an AUV waits for a neighboring AUV for a fixed period called the “*time-out interval*”. However, with the fixed time-out interval, it is highly probable that an AUV may miss the opportunity for synchronization with its neighbor owing to the variations in their mobility profiles. This results in the reduction of synchronization frequency that is the number of times an AUV synchronizes with its neighbors in a specific interval. This waiting increases not only the horizontal delay associated with the AUV→AUV communication but also the vertical delay associated with the node→AUV communication at the next tour-point. Even though the time-out interval is extended to be sufficiently long so that the synchronization with the neighboring AUV is not missed, the increase in the horizontal and vertical delay is unavoidable at the following tour-points. In addition, there may be an additional delay caused by an AUV waiting at the RP in case the neighbor has no data to forward. The aforementioned delays are inherent in a multiple AUV data-gathering scheme. Thus, the method of delivering time-critical data in a large-scale network employing multiple AUVs becomes significant.

In this paper, a data-gathering scheme using multiple AUVs is proposed to reduce the data-gathering latency over the large-scale hierarchical UWSN described in [8]. In the proposed scheme, all the AUVs share their status information via a selected node named the “*agent-node*”, to create a schedule for inter-AUV communication, minimizing both the vertical and horizontal delays. The AUV’s status information includes its mobility profile, operating mode (explained later), cumulative age of the packets in the buffer, and information regarding the neighboring AUV’s schedule. Unlike previous works where the AUV waits at a tour-point for the inter-AUV communication until a neighboring AUV arrives at its own tour-point, in the proposed scheme, the AUV may move forward to another stopping point named the *dynamic rendezvous point* (DRP) that is calculated in such a way that its arrival time at the DRP coincides with the arrival of the neighboring AUV at its own tour-point. Thus, removing the wasteful waiting time at the tour-point leads to a reduction in both the horizontal and vertical delays. In addition, to avoid a delay in the AUV synchronization, the proposed scheme does not require a time-out interval and speed control in contrast to the fixed-RP [16] and movement-scheduling [17], respectively.

The proposed scheme is evaluated in terms of the data transfer delays on the links of node-to-AUV (vertical), AUV-AUV (horizontal), and node-to-sink (end-to-end), AUV synchronization frequency, AUV tour-time, and the packet loss ratio comparing the following schemes:

1. Fixed-RP scheme [16]: With a time-out interval, an AUV waits for its neighboring AUV at a tour-point that is a fixed RP.
2. Global Relay AUV (GR-AUV) scheme [17]: An additional AUV not belonging to any specific region is employed only for collecting data from the local AUVs. The additional AUV visits each region and acts as a global relay.

The preliminary version of this work was presented in a conference paper [18]. This article is an extension of the conference version with additional details on the system description along with the network design and operation. The detail includes criteria for agent-node selection, AUV operating mode and DRP design. Similarly, additional results related to the vertical and horizontal delays, end-to-end delay, AUV synchronization frequency, AUV tour-time, and the packet loss ratio are included in the performance evaluation section of the paper. The rest of the paper is organized as follows: In Section 2, a summary on the related work is given. In Section 3, the system description is outlined. In Section 4, the operation of the proposed scheme is presented. The performance evaluation is presented in Section 5. Finally, Section 6 concludes this article.

## 2. Related Work

The networking of sensor nodes using multiple mobile nodes is extensively studied to improve the network lifetime and sensing coverage [19,20,21,22,23]. In these studies, it is assumed that the mobile nodes constitute a constantly connected network and acquisition of topology information is not an issue. Thus, a network can be reconfigured to provide full coverage. However, the implementation of these studies may be practically unrealistic to the adverse underwater environment where medium mobility greatly affects the inter-node communication and to overcome the effects may result in large control overhead.

For surveying and environment sampling, the authors [11,12,13,14] have explored the swarm and flocking algorithms for achieving coordination among multiple mobile nodes over the network. These algorithms also require a constant interconnection of a mobile node with its neighboring mobile nodes for an effective implementation. However, a constantly varying underwater channel leads to drastic changes in the communication probabilities over the network, rendering the constant interconnection of these mobile nodes unrealistic. Therefore, a direct application of these algorithms for the desired coordination, necessary for multihop communication among neighboring mobile nodes, may be difficult to achieve.

In [24,25], the authors have proposed data-gathering schemes using multiple mobile nodes, where first a set of data gathering locations called polling points are generated using spanning tree covering algorithm, then, a spanning tree is generated on the polling points. After that, the spanning tree is decomposed into several subtrees and a mobile node is assigned a subtree. Finally, the mobile node finds the shortest trajectory of polling points of subtree and starts data-gathering. After completion of data-gathering, the mobile node forwards the data to nearby mobile node so that the data can be offloaded to a mobile node that will visit the sink. In these schemes, it is also assumed that the mobile nodes are continuously connected and have the topological information on the neighboring mobile nodes. However, such assumption may not be valid in underwater environment where communication probabilities changes continuously owing to the topological changes and mobility of medium. Therefore, these schemes may not be operable in severe underwater conditions. In addition, the schemes also rely on precise movement control of mobile nodes for effective inter mobile node communication. Therefore, not anyone of the schemes may be a good candidate for implementation in underwater environment where variations in the underwater currents severely affect the mobile node mobility.

In [17] the authors have investigated the usage of multiple mobile nodes for data-gathering over a hierarchical network architecture, where mobile nodes are scheduled to meet each other at the synchronization points during certain time intervals. The proposed movement-scheduling scheme is vastly dependent on the mobility of the mobile nodes; therefore, it may be severely affected by the highly dynamic underwater environment. Similarly, the stringent requirement of movement control over the tour-path requires feedback information from the neighboring nodes that may not be readily available. Thus, the movement-scheduling approach may be difficult to implement.

To deliver time-critical data, authors in [26] proposed a scheme where multiple AUVs are deployed for data-gathering over the network. In this scheme, the trajectories for AUVs are planned using the value of information on the sensor nodes. The value of information is assigned to each packet and it decays with the passage of time. Every AUV visits a subset of sensor nodes on the assigned trajectory and then resurfaces to forward data to the base station. The proposed scheme maximizes the quality of data collection and balance the task of data collection among the AUVs. However, the global knowledge on the value of information on individual sensor node may be difficult to obtain in highly random underwater environment. Thus, such scheme may cause loss of critical information in real scenario.

For reporting the time-critical data, a data forwarding protocol is presented in [27], where the movement of AUVs deployed at the same trajectory is scheduled in such a way that the resurfacing and diving of AUVs can be controlled. The protocol assumes that the AUVs have the same speed, whether they are moving, surfacing or diving, for the trajectories scheduling of the AUVs. Even though, the proposed trajectory scheduling strategies reduce the data forwarding delay and control the frequency of diving and resurfacing, the constraint of same speed of AUVs make it impractical for real time underwater scenario.

In this paper, we have developed a mechanism for sharing the topological information among the deployed AUVs that reduces the delay in the multihop data forwarding among the AUVs. It reduces the delay by eliminating the time-out interval and unnecessary waiting at the tour-point that might result in missed synchronizations as in the case of [16]. In addition, unlike [17,24,25], the proposed scheme does not require any movement control of mobile nodes that is necessary for scheduling the neighboring mobile node to achieve effective multihop data forwarding.

## 3. System Description

### 3.1. Basic Assumptions

It is assumed that a numbers of acoustic sensor nodes are submerged at a certain depth in a given oceanic region for the purpose of data collection, monitoring, and surveying, as described in [28,29,30]. These sensor nodes are equipped with communication, data processing, and storage modules. The operational capacities of these modules are limited and they can only be operated for a specific duration owing to the limited onboard power resources. For a specific event sampling, these sensors nodes are also equipped with the relevant sensing module. The onboard communication module is capable of a discrete power control. Therefore, the sensor nodes can easily adjust their communication range. Each sensor node is assigned a unique address for its identification. The relevant information on the geographical location is provided to each sensor node at the time of its installation in the network.

It is also assumed that several battery-powered AUVs are available in the network and that they work as data mules. These vehicles are equipped with inertial and acoustic navigational tools [31]. An AUV utilizes the onboard navigational tools and specifically designed error correction algorithms to adjust its location in the network. Thus, every AUV transmits a periodic beacon message [32] to inform the network of its location. By adjusting its buoyancy, an AUV can control its depth in a given region. The AUV also have sufficient data storage, and highly sensitive receiving and processing modules to handle the incoming data traffic more efficiently than the sensor nodes in the network. Similarly, each AUV has a transmitting unit that is capable of establishing a reliable long-range forwarding link more accurately.

### 3.2. Network Architecture

**Figure 1:**
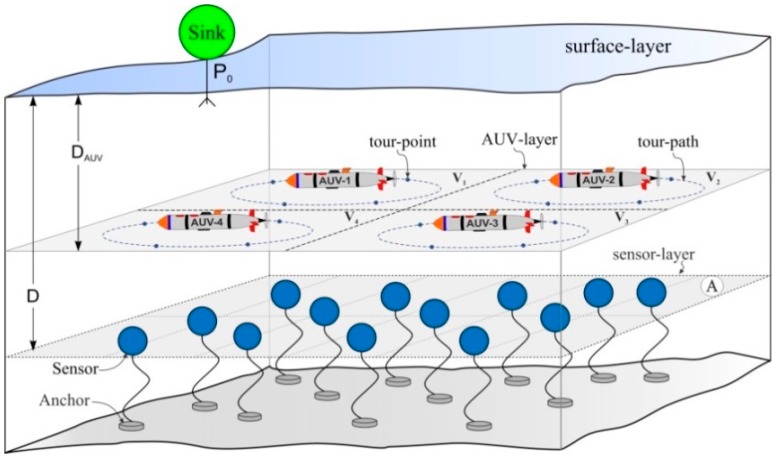
Proposed Network Architecture in a 3-dimensional region, *R*^3^.

The hierarchical underwater network described in [8,33] is considered; it consists of three layers, sensor-layer, AUV-layer, and surface-layer, as shown in Figure 1. The sensor-layer, where *N* sensor nodes are distributed uniformly, is at a depth, D, in a plane, A, of a given region, *R*^3^. These sensor nodes are anchored firmly to the ocean bottom and are assumed static. The AUV-layer is located in another plane at a depth, D_AUV_ < D and consists of $N_{v}$ AUVs. The surface-layer consists of a sink that is located at a position, **P**_0_.

The plane, A, is divided into several regions by means of the Voronoi region and generator-point concept, as described in [34]. One AUV is assigned to each region, where the AUV follows a specific trajectory called the *tour-path*. On the tour-path, the number of stopping locations named *tour-points* are predefined, where the AUV gathers data from the surrounding sensor nodes called the *member nodes* (MNs) belonging to the tour-point and then possibly forwards the data to the next-hop AUV. The data reception from a neighboring AUV takes place at the tour-point or another stopping point named the *dynamic rendezvous point* (DRP).

**Figure 2:**
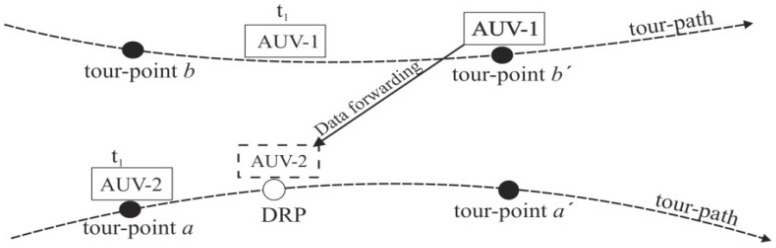
AUV→AUV data-gathering at the DRP.

Figure 2 shows the methodology by which data forwarding between the AUVs is achieved. Suppose AUV-1 is trying to forward data to AUV-2, in this case, we call AUV-1 and -2 as the child and parent AUVs, respectively. At a time instant, $t_{1}$, AUV-2 arrives at a tour-point, a; meanwhile AUV-1 is traveling from tour-point, b, to tour-point, b′. In the fixed-RP scheme [16], AUV-2 has to wait until AUV-1 arrives at the tour-point, b′. In the proposed scheme, if the estimated waiting time at the current tour-point, a, is larger than a predefined threshold, AUV-2 moves forward to a DRP which is designed in such a way that the time taken by AUV-2 to reach the DRP is same as the time taken by AUV-1 to reach tour-point, b′. This allows AUV-2 to establish a communication link with AUV-1 for data-gathering at the DRP instead of at the tour-point, a. Thus, AUV-2 utilizes the waiting time to move towards the DRP, reducing not only the vertical delay at the upcoming tour-points but also the horizontal delay at next tour-point, a′. However, if the estimated waiting time is lower than the threshold, then AUV-2 waits at the tour-point, a, until AUV-1 arrives at the tour-point, b′, and forwards the data.

To design a DRP, an AUV acquires the status information of the neighboring AUVs from a selected node named the *agent-node* that is assigned to every neighboring AUV, and is responsible for collecting and sharing the information of the respective AUV’s status. An AUV selects that MN as an agent-node that is closer to the agent-node of the neighboring AUV in adjacent cluster. The agent-node also acts as an MN. The tasks of the AUV are as follows:

1. It organizes the sensor nodes into a cluster around the generator point [35].
2. It constructs the tour-path that consists of a number of tour-points.
3. It divides the cluster into several subclusters by associating the MNs with the nearest respective tour-point.
4. It selects an agent-node and obtains the status information on the neighboring AUV. Based on this information, it schedules the data forwarding to the parent AUV or data-gathering from the child AUV.

### 3.3. Multiple Access Control

**Figure 3:**
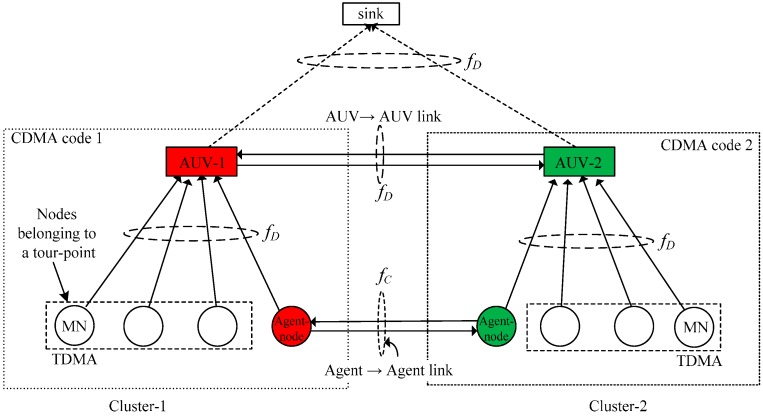
Medium-access scheme.

The communication channel, which refers to a frequency band here, is divided into two channels: the data channel, $f_{D}$ and the control channel, $f_{C}$. The data channel is used to forward data in the node→AUV, AUV→AUV, and the AUV→sink links, as shown in Figure 3. As the node→AUV and the AUV→AUV links are active at different instants of time, one channel can be used for both the links. Within a cluster, the data channel is exclusively allocated to the MNs associated with a tour-point by the AUV using a contention-free protocol such as the time-division multiple access (TDMA). Meanwhile, the control channel is shared by the local agent-nodes belonging to a cluster in a round robin manner to broadcast the AUVs’ status information. To avoid interference between adjacent clusters, the MN→AUV link is coded with an orthogonal code that is typically used in code division multiple access (CDMA) systems [36].

## 4. Operation of the Proposed Scheme

**Figure 4:**
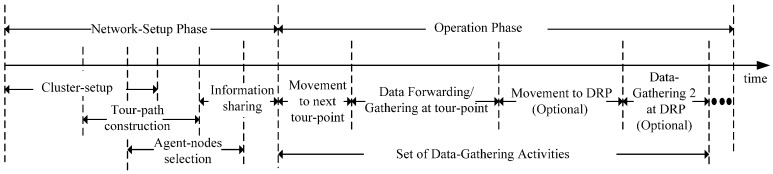
Timeline of the proposed scheme.

The proposed scheme runs through two phases, i.e., a network-setup phase and an operation phase, as shown in Figure 4. In the network-setup phase, first, an AUV moves to the Voronoi generator-point that is assigned by the sink and forms a cluster around the generator-point [34,35].

Then, the AUV calculates the tour-points in the cluster using the information on the MNs and constructs a tour-path (*Tour-path Construction*). Further, it selects an agent-node for every neighboring AUV (*Agent-nodes selection*). Finally, it exchanges the information regarding the tour-path and the local agent-nodes with the neighboring AUVs (*Information Sharing*) and enters an operation phase. In the operation phase, the AUV performs a set of sequential activities repeatedly. First, the AUV moves to the tour-point (*Movement to next tour-point*) and follows the *Data Forwarding/Gathering at tour-point* activity that includes data forwarding to the parent AUV, if the AUV is scheduled by the parent AUV; data-gathering from the MNs (*Data-Gathering 1*) and data-gathering from a child AUV (*Data-Gathering 2*), if the child AUV requires scheduling. If the estimated waiting time for Data-Gathering 2 from the child AUV is greater than a predefined threshold, then the AUV calculates a DRP and moves to the DRP (*Movement to DRP*) to gather data from the child AUV (*Data-Gathering 2 at DRP*).

### 4.1. Network-Setup Phase

First, the sink computes the Voronoi generator-points, ${\left\{z_{i}\right\}}_{i=1}^{N_{v}}$, using the procedure described in [34]. At this point, all the AUVs are docked at the location, **P**_0_, as shown in Figure 1. Before deploying the AUVs, the sink assigns the Voronoi generator-points and a distinct CDMA code to the respective AUVs. Then, the AUVs move to their respective generator-points and act as cluster-heads (CHs), as described in the cluster-setup phase of the LEACH protocol [37]. After stopping at the generator-point, the AUV broadcasts the network partitioning information (NPI) to the neighborhood. The nodes use the received NPI to determine the cluster to which they belong. After identifying the cluster, the sensor nodes reply to the corresponding AUV using the CSMA MAC protocol, becoming MNs. During this process, the AUV obtains the location of each MN in the cluster. Further, the AUV designs the tour-path to minimize the data-gathering time from the MNs.

#### 4.1.1. Tour-Path Construction

After the cluster-setup, the AUV uses the procedure described in [38] to construct the tour-path. In this procedure, the AUV virtually draws circles with the same radius, *r*, at the individual MNs’ locations. Varying, *r*, the AUV determines certain points where a group of circles overlap. These points act as the candidate tour-points and the corresponding group of MNs forms a subcluster. Then, the AUV randomly selects a tour-point among the candidate ones of each subcluster and connects them to form tour-path [39]. Even though the obtained tour-path is not an optimal solution, it can work to reduce the total data-gathering time from the MNs, minimizing the variance of the data-gathering time of each sensor node. The design of the optimal tour-path is beyond the scope of this paper and is considered as future work.

#### 4.1.2. Agent-Node Selection

In the absence of constant communication among the AUVs, it is difficult for an AUV to obtain the latest status information of its neighboring AUVs. Therefore, after designing the tour-path, an AUV selects agent-nodes that collect the status information of their respective neighboring AUVs. For example, let AUV-*i* have two neighboring AUVs, AUV-*j* and AUV-*k*; then two agent-nodes, $X_{i↔j}$ and $X_{i↔k}$, are selected by AUV-*i* to share the status information with the counterpart neighboring agent-nodes, $X_{j↔i}$ and $X_{k↔i}$, respectively. The agent-node, $X_{i↔j}$, is selected as the closest one to the generator point, $z_{j}$, among the MNs satisfying the following conditions:

- The residual energy should be larger than a given threshold, ε*_th_*.
- The distance to the counterpart agent-node here, $X_{j↔i}$, should be shorter than a given threshold, δ*_th_*.

Periodically broadcasting the respective AUV’s status information, the agent-nodes expend more energy than the normal MNs. Thus, constraint (ii) helps in reducing the energy consumption by limiting the communication link distance. As the tour progresses, the residual energy of the agent-node may decrease below ε*_th_*. Then, the AUV reselects a new agent-node to satisfy the aforementioned conditions. To do this, the AUV needs to continuously monitor the residual energy of the agent-node as well as the location of the neighboring agent-nodes.

**Figure 5:**
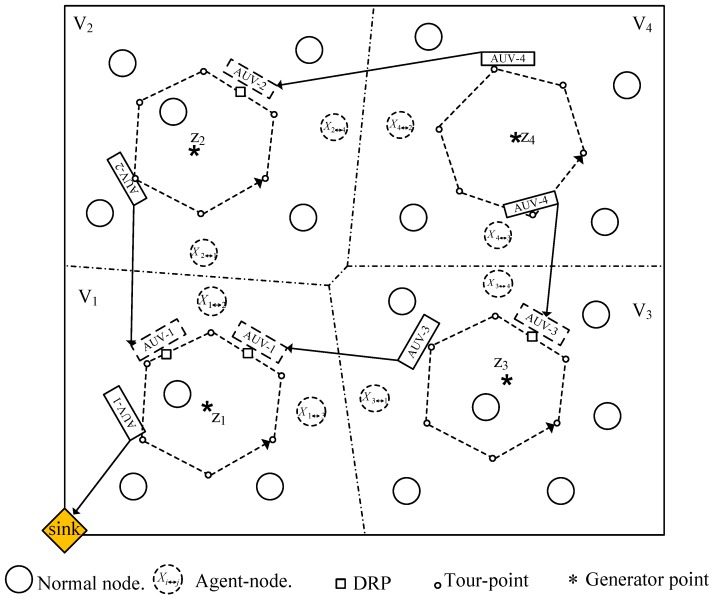
Acoustic Sensor Network with Voronoi regions.

Figure 5 shows an example of the proposed network, where the entire area is divided into four clusters corresponding to the Voronoi regions ($V_{i}$, *i* = 1, 2, 3, 4) generated by the Voronoi generator points ($z_{i}$, *i* = 1, 2, 3, 4). The dashed-rectangles and solid-rectangles represent the AUVs in the receiving and forwarding states, respectively.

#### 4.1.3. Information Sharing

After the selection of the agent-nodes, the AUVs construct a data-forwarding tree to the sink on the AUV-layer by exchanging relevant information based on the distance-vector routing algorithm [40]. After construction of the data forwarding tree, the AUVs exchange the information on their tour-paths and the location of the agent-nodes.

### 4.2. Operation Phase

Before discussing the details of the operation phase, the operating mode of an AUV is described.

#### 4.2.1. AUV Operating Mode

Two different modes are defined for an AUV operation:

1. Mode-r (receiving): Preparation for gathering data from a child AUV.
2. Mode-f (forwarding): Preparation for forwarding data to a parent AUV.

**Figure 6:**
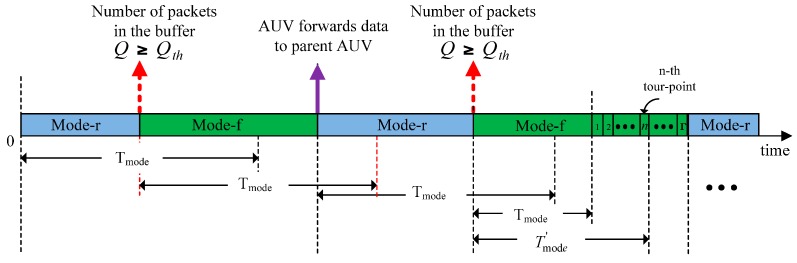
AUV operating mode transition diagram.

The duration of both the modes are commonly predefined by T_mode_. However, under certain conditions, the modes can be switched before the expiration of T_mode_ or can be continued to exceed T_mode_. T_mode_ is chosen to be long enough such that an AUV collects sufficient data to forward but not too long such that the delay requirement is satisfied. Let *Q* denote the number of packets in the buffer of an AUV at any instant. If *Q* exceeds a threshold, *Q_th_*, then, the AUV switches the mode from mode-r to –f before T_mode_ has expired (denoted by a dotted arrow in the Figure 6).

Switching to mode-f, the AUV becomes a child. Then, the child AUV notifies the mode transition to the agent-nodes so that it can be scheduled by a parent AUV. Further, at each upcoming tour-point, the child AUV checks with the agent-nodes to determine if it is scheduled by the parent AUV. Meanwhile, a parent AUV also uses the agent-nodes to obtain the status information of the child AUVs. The parent AUV schedules a child AUV if it finds the child AUV in mode-f. Then, the parent AUV updates the child AUV scheduling information with the agent-nodes so that the child AUV can forward data at an upcoming tour-point. After completing the data forwarding to the parent AUV, the child AUV goes back to mode-r, denoted by a solid arrow and this may occur before the expiration of T_mode_, as shown in Figure 6. Now, the child AUV becomes a parent AUV to another AUV.

However, if a child AUV fails to be scheduled for data forwarding within the T_mode_, mode-f is extended until the AUV arrives at the next tour-point. Such an extension is allowed to continue up to the number of Γ, beyond which the child AUV is forced into mode-r. During the extended period (*T*’_mode_), at each upcoming tour-point, the child AUV checks its scheduling status with the agent-node. If the child AUV is scheduled, it forwards the data to the parent AUV and enters mode-r.

#### 4.2.2. Movement

Moving towards a tour-point, an AUV periodically broadcasts a beacon message so that the agent-nodes update the AUV’s status information [32,41] containing the operating mode status, cumulative age of the data packets in the buffer (defined later), current location, information on the last visited tour-point, and the upcoming tour-point. Then, the agent-nodes exchange the AUV’s status information with the neighboring agent-nodes.

#### 4.2.3. Data Forwarding/Gathering at the Tour-Point

##### In Mode-f

Arriving at a tour-point in mode-f, an AUV communicates with the agent-nodes to check if it is scheduled for data forwarding by the parent AUV. If it is scheduled, then it sets the communication link with the parent AUV using handshaking [40] in the channel, $f_{D}$. It then forwards the data to the parent AUV.

**Figure 7:**
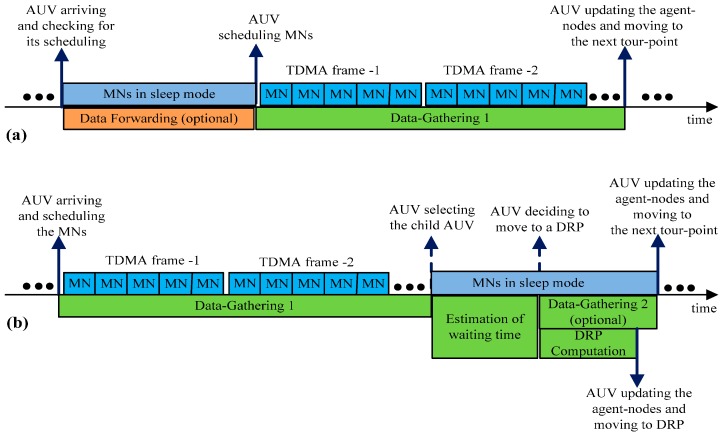
Events that may occur at a tour-point (**a**) AUV in mode-f (**b**) AUV in mode-r.

On the other hand, if the AUV is not scheduled, it immediately starts data-gathering from the MNs (Data-Gathering 1) skipping the data forwarding step. For this reason, the data forwarding is depicted as optional in Figure 7a. To perform Data-Gathering 1, the AUV broadcasts a beacon message containing the scheduling information for the MNs. On receiving the beacon message, the MNs wake-up and begin to transmit data packets in their allocated time-slots. During this process, the agent-nodes continue broadcasting and receiving the AUVs’ status information on the channel, $f_{C}$.

After completing the data-gathering from the MNs, the AUV calculates the cumulative age of the data packets, defined as,

(1) $$A=\sum_{k=1}^{Q}\alpha_{k}$$

where $\alpha_{k}$ is the age of the k-th packet in the buffer. The packet age is defined by the elapsed time from the packet generation instant, denoted in the packet. The AUV then informs the cumulative age of the data packets to the agent-node along with the other status information mentioned in the previous section. Then, the AUV moves to the next tour-point.

##### In Mode-r

Figure 7 b shows the sequential activities that the AUV performs when it arrives at a tour-point in mode-r. It starts Data-Gathering 1 in the same manner as it does in mode-f. After Data-Gathering 1, the AUV communicates with the agent-nodes to obtain the latest status information on the child AUVs. Among the child AUVs in mode-f, the AUV selects one that has the largest cumulative age of the data packets for scheduling.

After the selection of a child AUV, the AUV updates the agent-nodes with the child AUV’s scheduling information. In this process, the AUV also obtains the child AUV’s latest mobility profile in order to estimate the waiting time of the child AUV. If the estimated waiting time is less than τ, the maximum allowable waiting time, then, the AUV waits at the current tour-point until the child AUV reaches its tour-point and performs Data-Gathering 2. However, if the estimated waiting time is greater than τ, the AUV moves to a DRP for Data-Gathering 2.

##### DRP Computation

**Figure 8:**
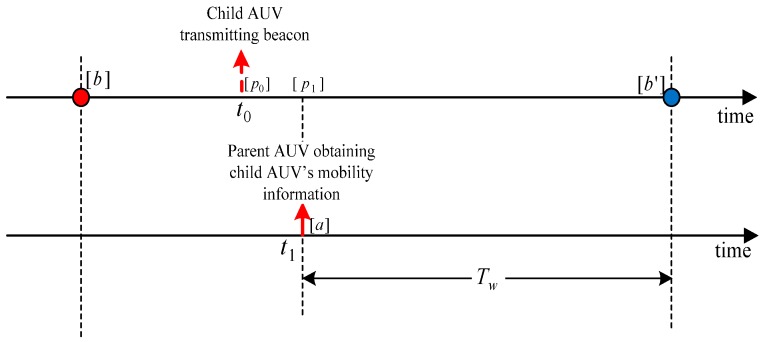
Graphical representation of the parent AUV’s estimated waiting time, $T_{w}$ (All the locations are denoted within square brackets).

Figure 8 shows an example wherein the child AUV is moving from tour-point b to b′ with an average velocity, $v_{c}$ and the parent AUV waiting at a tour-point (say a) obtains at a time, $t_{1}$, the latest mobility profile of the child AUV that is transmitted at time, $t_{0}$ and at a position, $p_{0}$. Then, the position of the child AUV at time, $t_{1}$, is estimated by,

(2) $$p_{1}=p_{0}+\left(t_{1}-t_{0}\right)\cdot v_{c}$$

which is subject to the operation of a vector summation and a scalar multiplication to the vector. Accordingly, the estimated waiting time is obtained by,

(3) $$T_{w}=\frac{\left\|b^{\prime }-p_{1}\right\|}{\left\|v_{c}\right\|}$$

where ‖·‖ represents the vector norm. As mentioned earlier, if $T_{w}$ is less than τ, then the parent AUV waits at the tour-point, a; otherwise, it calculates the DRP as,

(4) $$DRP=a+T_{w}\cdot v_{p}$$

where $v_{p}$ is the average velocity of the parent AUV. Before moving to the DRP, the parent AUV notifies the location of the DRP to the agent-nodes.

#### 4.2.4. Data-Gathering 2 at the DRP

Arriving at the DRP, the AUV waits until the child AUV establishes a communication link for data forwarding. After the completion of Data-Gathering 2, the AUV updates the status information with the agent-nodes and starts moving to the next tour-point.

## 5. Performance Evaluation

### 5.1. Simulation Setup

We have developed a discrete event-driven network simulator using MATLAB (Mathworks, Natick, MA, USA) to evaluate the proposed scheme. We have considered a network, where 100 sensor nodes are uniformly distributed at the average depth of 300 m in an area of size 5 × 5 km^2^. We have modeled the acoustic channel attenuation and noise as described in [42]. In addition, the nominal speed of the acoustic signal is assumed to be 1500 m/s. We consider that every sensor node and AUV is equipped with a WHOI micro-modem-2 [43]. Therefore, we have set the data transmission parameters for the sensor nodes and AUVs accordingly, and they are listed in Table 1. It is assumed that the modem installed on an AUV is built using a high-end Blackfin processor that provides sufficient processing power to handle the large number of iterations required for a tour-path construction in the proposed networking scenario. It is probable that multiple events of interest may happen simultaneously in the proximity of a sensor node that may lead to a higher traffic load. Therefore, the memory block at modem is enhanced so that the data storage at the sensor nodes may take place efficiently before delivery to the local AUV. Further, it is assumed that a Poisson traffic is generated at every sensor node in the network.

It is assumed that the AUVs are operating in their respective tour-paths at an average depth of 250 m. We have modeled the variations in the AUV velocity using a discrete Gauss-Markov Model [16,44]. The memory level of the Gauss-Markov model is set to 0.5 to obtain an AUV velocity with a mean of 2 m/s and a variance of 0.5.

The network is partitioned into four Voronoi regions using the procedure described in [34]. We have compared the performance of the proposed scheme with the fixed-RP scheme [16] and the GR-AUV scheme [17]. For a fair comparison of the three candidate schemes, the same network topology with an equal number of clusters and AUV tour-paths has been used. In addition, we have used Voronoi generator points to simulate the tour-path of the GR-AUV.

### 5.2. Performance Metrics

Following metrics are used to analyze the performance of the proposed scheme.

- *AUV synchronization frequency*: the number of times an AUV synchronizes with its neighbors in a specific time interval.
- *Average horizontal delay*: the average time spent by a packet from the time it arrives at a child AUV to the time it is delivered to a parent AUV.
- *Average vertical delay*: the average time spent by a packet from the time it is generated at a node to the time it is delivered to an AUV.
- *End-to-end Delay*: the time spent by a packet from generation to delivery to the sink.
- *AUV tour-time*: the time taken by an AUV to completely traverse its tour-path.
- *Packet loss ratio (PLR)*: the ratio of the number of arrived packets at the sink with a delay larger than the delay threshold to the number of generated packets.

### 5.3. Performance Analysis

**Figure 9:**
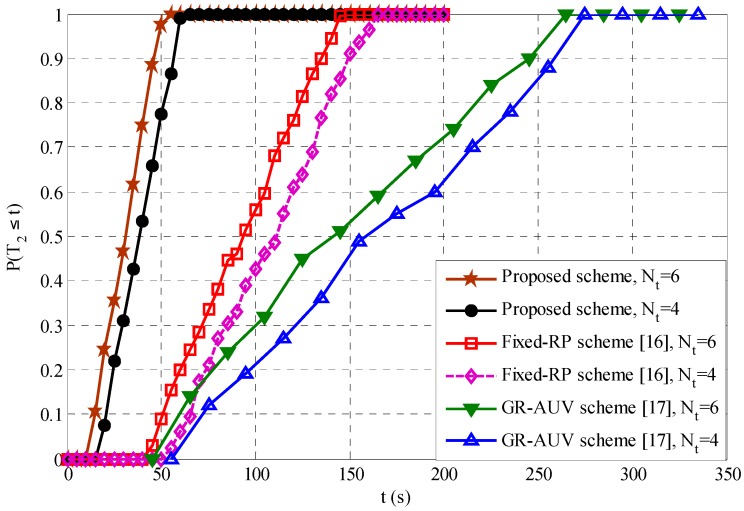
Cumulative distribution Probability of $T_{2}$.

In Data-Gathering 2, the AUV waits until a child AUV arrives at the tour-point and the communication link is established. Let such a waiting time for Data-Gathering 2 be $T_{2}$. Figure 9 shows the cumulative distribution probability of $T_{2},P\left(T_{2}\leq t\right)$, varying the number of tour-points, $N_{t}$, for a mean inter-node distance of 400 m.

The simulation results clearly indicate that the $T_{2}$ of the proposed scheme is lower than the fixed-RP scheme and the GR-AUV scheme. This is because unlike the fixed-RP scheme, where the AUV waits until a child AUV reaches the tour-points or the time-out interval has expired, in the proposed scheme, an AUV can reduce the waiting time by moving forward to the DRP, while the child AUV is travelling to the tour-point. In the GR-AUV scheme, as a time-out interval is not employed, the GR-AUV has to wait unexpectedly long until the child AUV arrives, resulting in larger waiting time. For example, it may be owing to the possibility of the GR-AUV arriving at the tour-point, when the child AUV has just started moving towards the next tour-point and has missed the beacon message from the GR-AUV. It is also shown that $T_{2}$ for all the schemes decreases as the number of tour-points increase. This is because the increase in the number of tour-points increases the possibilities of the parent and child AUVs being-connected at their designated tour-points with minimal waiting times.

**Figure 10:**
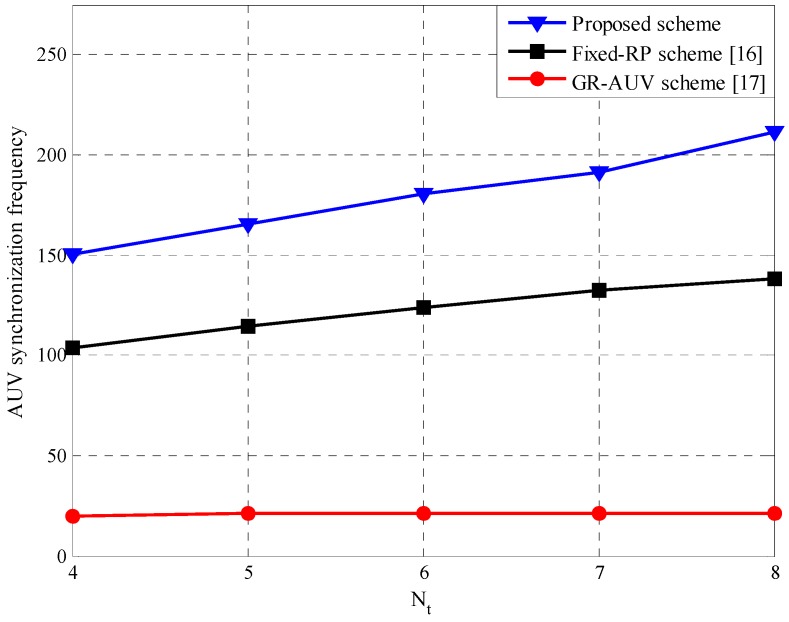
AUV synchronization frequency versus the number of our-points, $N_{t}$.

A parent AUV is synchronized with child AUV several times during a tour. Less synchronization implies less opportunities for data-gathering/forwarding, thereby, increasing the packet loss or delay. Figure 10 shows the AUV synchronization frequency, the number of synchronizations over a specific duration of $10^{5}$ s. The proposed scheme has a higher synchronization frequency than the fixed-RP and GR-AUV schemes because a parent AUV is able to reduce the waiting time based on the status information of a child AUV and accordingly has more chances for data-gathering, while in the case of the fixed-RP scheme, a parent AUV may miss the synchronization owing to a time-out expiration; in the GR-AUV scheme, the long travel of the GR-AUV results in a long time for the synchronization.

**Figure 11:**
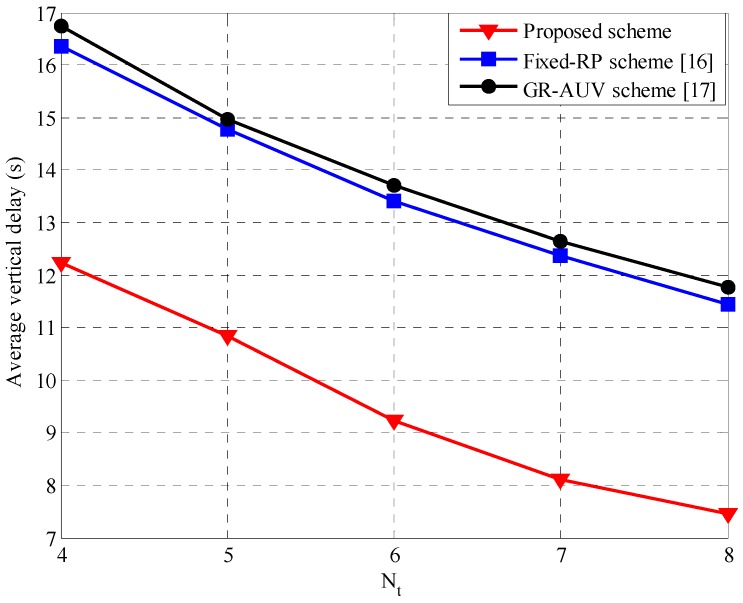
Average vertical delay versus the number of tour-points, $N_{t}$.

Figure 11 shows the average vertical delay varying the number of tour-points, $N_{t}$. The proposed scheme has a lower vertical delay than the fixed-RP scheme and the GR-AUV scheme because an AUV, not waiting at the tour-point, moves forward covering the additional tour-path, thereby, reducing the delay for the packets associated with the subsequent tour-points. On the other hand, in the fixed-RP scheme, the parent AUV has to wait unnecessarily for a child AUV, even when sufficient data packets are not available for gathering. The GR-AUV scheme that has a similar Data-Gathering 1 procedure as the fixed-RP scheme, has a slightly larger vertical delay than the fixed-RP scheme because the child AUV checks the GR-AUV arrival at every tour-point causing additional delay for data associated with the next tour-points. In addition, as a child AUV is scheduled once during the entire tour of the GR-AUV, it takes a longer time for the child AUV to empty its buffer at a tour-point; accordingly, the vertical delay associated with the next tour-point increases. The reason for the decrease in the vertical delay for a larger number of tour-points is same as that discussed in Figure 9.

**Figure 12:**
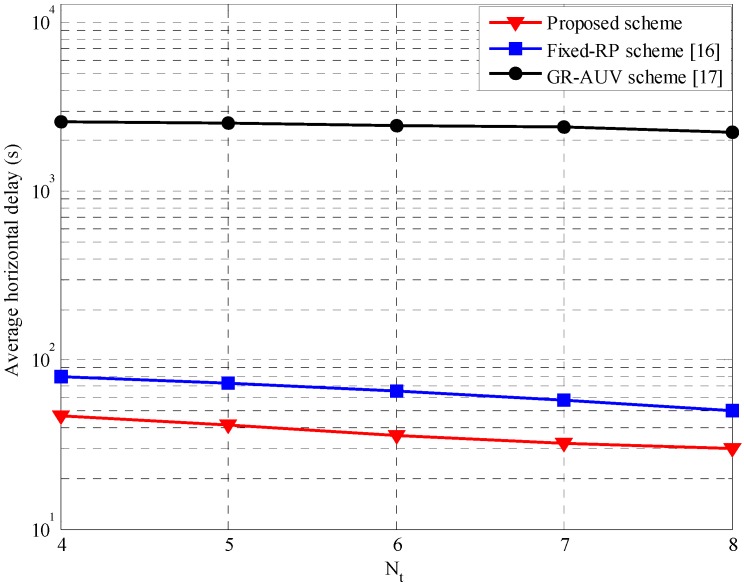
Average horizontal delay versus the number of tour-points, $N_{t}$.

Figure 12 shows the average horizontal delay performance of the proposed scheme in comparison to other candidate schemes varying the number of tour-points, $N_{t}$. It is observed that the average horizontal delay for the proposed scheme is lower than that of the fixed-RP scheme and the GR-AUV scheme. In the proposed scheme because a parent AUV uses the status information of a child AUV to build an alternative data-gathering point, DRP, in the process of reducing the waiting time, the possibility of the child AUV missing the opportunity for data forwarding is significantly reduced. Thus, the stored data at the child AUV is delivered on time to the parent AUV. On the other hand, in the fixed-RP scheme, a child AUV may miss the opportunity for forwarding data to a parent AUV owing to a time-out causing the parent AUV to depart to the next tour-point. In the GR-AUV scheme, the longer traveling time of the GR-AUV results in a large horizontal delay.

**Figure 13:**
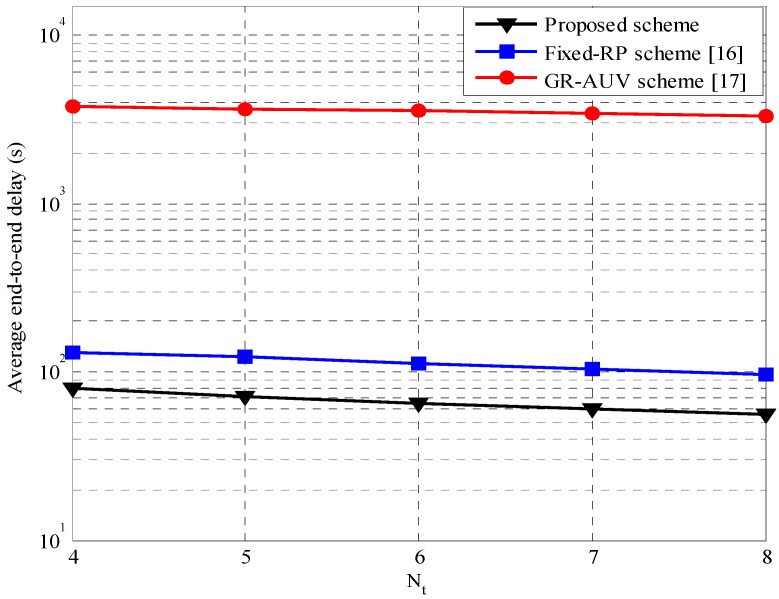
Average end-to-end delay versus the number of tour-points, $N_{t}$.

Figure 13 shows the average end-to-end delay, which is the sum of the horizontal and vertical delays. The end-to-end delay of the proposed scheme is lower than that of the fixed-RP and GR-AUV schemes owing to the same reason described for the horizontal and vertical delays. Even though the proposed scheme may generate control traffic associated with the agent nodes, as the control traffic is carried by a separate control channel, the end-to-end delay that occurs on the data channel is not affected.

**Figure 14:**
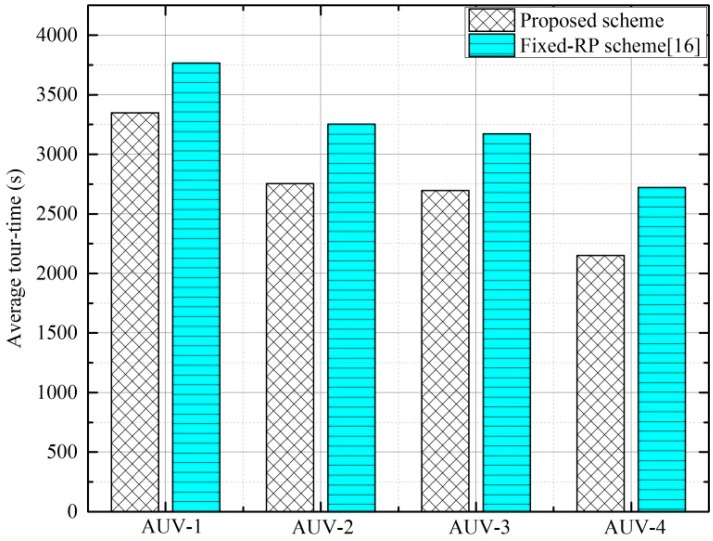
Average tour-time.

We also explore the effect of an AUV’s location in the hierarchical tree topology on its tour-time. For this purpose, the scenario shown in the Figure 5 is considered, where AUV-1 has two child AUVs (AUV-2 and -3) and AUV-4 acts as a child to both AUVs-2 and -3. Figure 14 shows the average tour-time of the proposed scheme and the fixed-RP scheme. As the GR-AUV has an obviously larger tour-time than the others, this scheme is not considered. The average tour-time of AUV-1 is larger than the other AUVs because AUV-1 spends time in scheduling more child AUVs, possibly waiting more at the tour-points and stopping at more DRPs. On the other hand, AUVs-2 and -3 have almost the same average tour-time owing to the same location from the topology point of view and one common child AUV. As they share the traffic load of AUV-4, the data gathering time from AUV-4 is also reduced.

**Figure 15:**
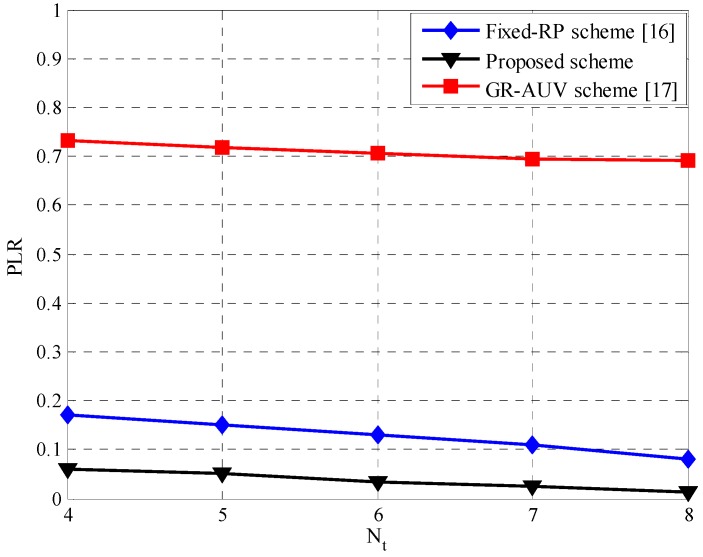
Packet loss ratio versus the number of tour-points, $N_{t}$.

Most time-critical applications require data to be delivered to the sink within an acceptable delay so that the data may be utilized effectively. To determine the effectiveness of proposed scheme for such applications, we compare the PLR performance of all schemes. Figure 15 shows the PLR with the delay a threshold of 540 s. It is observed that the GR-AUV scheme has the worst PLR because the GR-AUV has not only a longer traveling time but also a longer waiting time at the tour-points owing to the lack of a time-out interval. The reason why the PLR of the proposed scheme is better than the fixed-RP scheme is because employing a DRP to gather data from a child AUV can significantly reduce the delay for the data packets.

**Figure 16:**
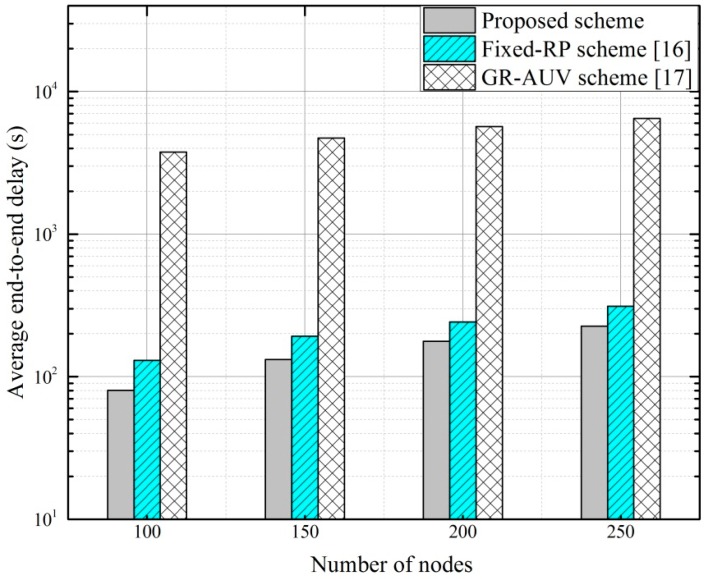
Average end-to-end delay versus the number of nodes.

Figure 16 shows the end-to-end delay performance of the candidate schemes for four different node deployment scenarios. The mean inter-node distance in each scenario is considered to be 400 m. In every scenario, a network is partitioned into several clusters in such a way that a cluster has an average of 25 sensor nodes. With increase in the number of sensor nodes, the size of the data forwarding tree at AUV-layer increases not only for the fixed-RP scheme but also for the proposed scheme. This results in the increase of end-to-end delay for both schemes. Since the increase in the number of nodes increases the number of clusters in the network, the length of the GR-AUV tour become large that results in higher end-to-end delay for GR-AUV scheme. However, in all node deployment scenarios, the end-to-end delay for proposed scheme remains less than the other schemes owing to the sharing of AUVs’ status information.

## 6. Conclusions

In this paper, we have presented a multihop data-gathering scheme using multiple AUVs over a large-scale hierarchical UWSN deployed for an environmental monitoring application that requires sensor data to be delivered to a sink within acceptable latency limits. In the proposed scheme, multihop data forwarding is achieved through the AUVs’ synchronization that is severely affected by the varying underwater conditions. To achieve this, a parent AUV uses agent-nodes to obtain the latest information on the child AUVs. Then, it calculates a DRP and moves to the DRP for data gathering from a child AUV instead of waiting at the tour-point, unlike the fixed-RP scheme. Thus, a parent AUV utilizes the waiting time to cover an additional tour-path, reducing the delay for the data. The simulation results show that the end-to-end delay performance of the proposed scheme is lower than that of the fixed-RP and the GR-AUV schemes. In addition, the proposed scheme also outperforms both the fixed-RP and GR-AUV schemes in terms of the packet loss ratio, the AUV synchronization frequency, and the tour-time. Even though the usage of agent-nodes generates additional overhead in the proposed scheme, the reduction in end-to-end delay compensates for the overheads. This renders it a suitable candidate for implementation over a large-scale UWSN.

For a large-scale underwater networking scenario, where the AUVs’ synchronization is used to achieve multihop communication for delivering data to a sink, the realization of a scheme that achieves an optimal AUV synchronization is a challenging task because of the absence of information on the AUVs’ mobility parameters. The varying communication probability and constantly changing network topology at the AUV layer also makes it difficult to achieve the same. The proposed scheme is robust in the sense that it uses the agent-nodes to share the AUVs’ status information locally in a neighborhood, thereby, eliminating the requirement to disseminate such information globally for the maintenance of the data forwarding route. Thus, the proposed scheme is expected to achieve efficient multihop communication among the AUVs in a real underwater scenario.

In future, we propose to extend the proposed scheme for the selection of optimal agent-nodes based on the expected variation in the energy resources of the agent-nodes over the time duration required for an AUV to cover the distance between two consecutive tour-points. In addition, an adaptive tour selection algorithm will be developed so that the topological changes caused by either the failure of the nodes or the movements of the nodes can be controlled. We also expect to design a distributed flow control algorithm for optimizing the tour-times of all the AUVs.

## Figures and Tables

**Table 1:** Simulation Parameters.

Parameter	Value
Data Rate	2500 bps
Data packet length	1024 bits
Control packet length	256 bits
Center frequencies of $f_{C}$ and $f_{D}$	20 kHz. 30 kHz
AUV waiting-time threshold, τ	120 s
Time-out interval	240 s
